# Global transcriptome and coexpression network analyses reveal cultivar-specific molecular signatures associated with different rooting depth responses to drought stress in potato

**DOI:** 10.3389/fpls.2022.1007866

**Published:** 2022-10-19

**Authors:** Tianyuan Qin, Kazim Ali, Yihao Wang, Richard Dormatey, Panfeng Yao, Zhenzhen Bi, Yuhui Liu, Chao Sun, Jiangping Bai

**Affiliations:** ^1^ State Key Laboratory of Aridland Crop Science, College of Agronomy, Gansu Agricultural University, Lanzhou, China; ^2^ National Institute for Genomics and Advanced Biotechnology, National Agricultural Research Centre, Islamabad, Pakistan

**Keywords:** potato, coexpression network, gene expression, root development, rooting depth, transcriptional modules

## Abstract

Potato is one of the most important vegetable crops worldwide. Its growth, development and ultimately yield is hindered by drought stress condition. Breeding and selection of deep-rooted and drought-tolerant potato varieties has become a prime approach for improving the yield and quality of potato (*Solanum tuberosum* L.) in arid and semiarid areas. A comprehensive understanding of root development-related genes has enabled scientists to formulate strategies to incorporate them into breeding to improve complex agronomic traits and provide opportunities for the development of stress tolerant germplasm. Root response to drought stress is an intricate process regulated through complex transcriptional regulatory network. To understand the rooting depth and molecular mechanism, regulating root response to drought stress in potato, transcriptome dynamics of roots at different stages of drought stress were analyzed in deep (C119) and shallow-rooted (C16) cultivars. Stage-specific expression was observed for a significant proportion of genes in each cultivar and it was inferred that as compared to C16 (shallow-rooted), approximately half of the genes were differentially expressed in deep-rooted cultivar (C119). In C16 and C119, 11 and 14 coexpressed gene modules, respectively, were significantly associated with physiological traits under drought stress. In a comparative analysis, some modules were different between the two cultivars and were associated with differential response to specific drought stress stage. Transcriptional regulatory networks were constructed, and key components determining rooting depth were identified. Through the results, we found that rooting depth (shallow vs deep) was largely determined by plant-type, cell wall organization or biogenesis, hemicellulose metabolic process, and polysaccharide metabolic process. In addition, candidate genes responding to drought stress were identified in deep (C119) and shallow (C16) rooted potato varieties. The results of this study will be a valuable source for further investigations on the role of candidate gene(s) that affect rooting depth and drought tolerance mechanisms in potato.

## Introduction

Roots provide plants with water and nutrients. Root depth (deep or shallow) is an important agronomic trait related to yield potential and is particularly important in potato tubers (Xu et al., 2017; Wang et al., 2020b). In potato tubers, root development begins at tuber germination, and the first young buds appear with scaly or “embryo” leaves at the top, followed by young roots on several sections at the base of the buds (Nicolas et al., 2022). The early period is primarily for root formation and bud growth, which form the basis for tuber seedling rooting, tuber formation, and plant strengthening (Zhou et al., 2017; Omary et al., 2022). A comprehensive understanding of the molecular mechanisms regulating all aspects of root development is needed to facilitate the development of new cultivars.

Many biological processes and pathways control root development (Sheng et al., 2017; Zhao et al., 2020). Omics studies in model plants and crops provide molecular insights into gene pathways and regulatory networks and their interactions involved in various aspects of root development (Wang et al., 2020a; Chen et al., 2022). The molecular mechanisms controlling root depth (deep or shallow) have also been elucidated to some extent in some plants, such as *Arabidopsis* and rice (Jean-Baptiste et al., 2019; Maurel and Nacry, 2020; Reynoso et al., 2022). In the early stage of root development and carbohydrate partitioning, the supply of photoassimilates and the accumulation rate of storage substances are very important in determining rooting depth (Chai and Schachtman, 2022; Li et al., 2022). In addition, epigenetic mechanisms and hormone signalling have also been identified as important regulatory mechanisms that determine rooting depth (Gutierrez et al., 2009; Holzwart et al., 2018; Jia et al., 2019). Furthermore, the number of cells in Gramineae, including rice and maize, is positively correlated with rooting (Liu et al., 2021; Marand et al., 2021; Wang et al., 2021a).

Potato is a nutritionally and agriculturally important tuber crop that is commonly grown as a staple crop in arid and semiarid regions with an average annual rainfall less than 500 mm (Su et al., 2020). In these regions, potato tuber yield and quality s are constrained by numerous biotic and abiotic stresses, including prolonged or seasonal drought stress, which adversely affects canopy growth and tuber yield and market value (Fernie and Willmitzer, 2002; Barnaby et al., 2019). Access to potato transcriptomes and draft genome sequences (Zhou et al., 2020a; Sun et al., 2022) and next-generation NGS provide opportunities to uncover genetic diversity among different genotypes or cultivars, especially for important agronomic traits. Transcriptome studies have examined flower and leaf development and responses to abiotic stresses (Singh et al., 2013; Kudapa et al., 2014; Garg et al., 2016). However, similar WGCNA analysis method have not been conducted to determine the molecular mechanisms underlying root responses to drought stress or rooting depth in potato (Jain et al., 2017; Ma et al., 2021). Rooting depth (shallow or deep) is an important trait that influences plant response to abiotic stress (Vanhees et al., 2020). Although huge variations in rooting depth have been observed among potato genotypes, this phenotypic variability has not been used to improve rooting depth and to improve potato drought resistance of important potato cultivars. Deep-rooted genes in potato has not been exploited because the molecular mechanisms controlling rooting depth are poorly understood.

The availability of genotypes and cultivars with opposite phenotypes of specific traits provides an excellent opportunity to uncover the genetic factors controlling rooting depth. However, comparative transcriptome analysis of potato genotypes and cultivars with different rooting depths has not yet been performed. Therefore, in this study, RNA-seq technology was used to analyze the transcriptomes of roots of two potato cultivars that differed significantly in rooting depth (one deep-rooted and one shallow-rooted) at different stages of root response to drought stress. Data were analyzed to reveal transcriptome dynamics and transcriptional networks associated with root response to drought stress and to identify key genetic differences between deep-rooted and shallow-rooted potato cultivars in response to drought stress. Transcripts and modules of co-expressed genes that are predominantly or specifically expressed at different stages of root response to drought stress or in a particular cultivar were identified. The results of this study shed light on the molecular mechanisms that determine rooting depth and root response to drought stress in potato.

## Results

### Global transcriptome analysis of potato cultivar roots

**Figure 1:**
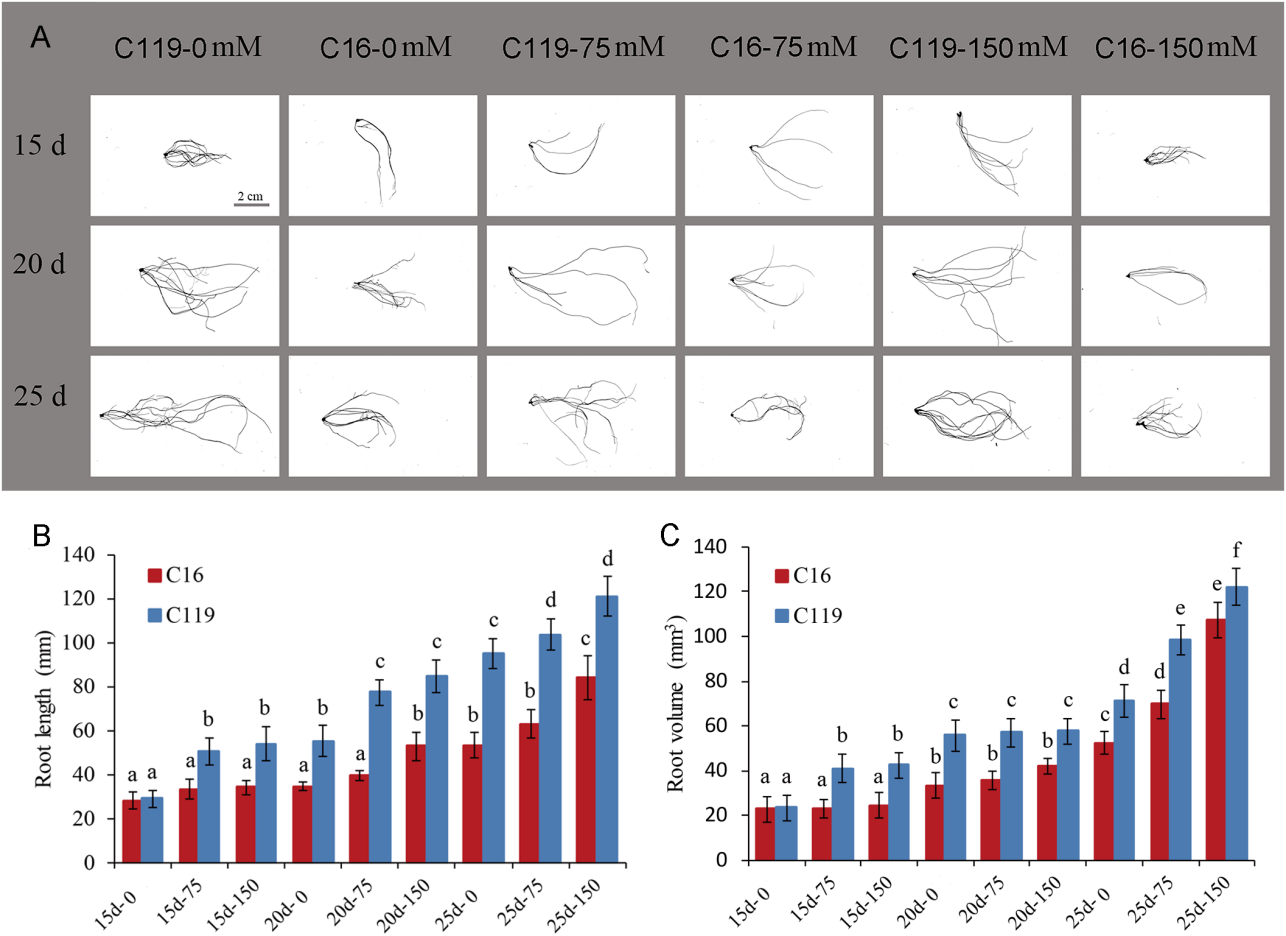
Root phenotypes at different stages of drought stress in potato cultivars C16 and C119. **(A)** Root phenotype at different stages of drought stress in C16 and C119. **(B, C)** Physical measurements showing variation in root length **(B)** and root volume **(C)** of C16 and C119 at different stages of drought stress. Root length (in mm) and root volume (in mm3) were plotted. The error bars indicate the standard error. The red bar represents C16, and the blue bar represents C119. The 15d, 20d and 25d represent the growth days; 0,75 and 150 represent the mannitol concentration.

To gain insights into the molecular mechanisms underlying root response to drought stress and controlling root length and volume. Root transcriptomes at different stages of root response to drought stress were analyzed in potato cultivars with differences in root length and volume (**Figure 1**, **Figure S1**). In this study, potato cultivar C16 was shallow-rooted and cultivar C119 was deep-rooted, and root length and volume of the two cultivars differed significantly. Nine stages of root responses to drought stress, representing important events within the root occurred (**Figure 1**). During the 15- to 25-day growth period, the average root length of C16 increased from 28 mm to 84 mm, while the average root length of C119 increased from 29 mm to 121 mm (**Figure 1** **B**). The average root volume of C16 increased from 22 mm3 to 107 mm3, while the average root volume of C119 increased from 23 mm3 to 122 mm3 (**Figure 1** **C**).

To investigate transcriptome dynamics during root response to drought stress, RNA-seq experiments were perform using total RNA from the three stages of root response to drought stress of potato cultivars C16 and C119. Three biological replicates of all samples were analyzed (54 samples in total). Each cultivar produced more than 0.6 billion high-quality reads (an average of about 23 million reads per sample) at different stages (**Table S1**). Reads were mapped to the potato genome (DM v4.04, http://spuddb.uga.edu/pgsc_download.shtml) using TopHat (Trapnell et al., 2012). The mapped files were processed using Cufflinks (Wang, 2018) and Cuffmerge (Li et al., 2016). which generated a consensus transcriptome assembly with a total of 40337 gene loci. Total mapped ratio of each sample (**Table S1**) were processed with Cufflinks to determine the standardized expression level of each transcript, i.e., transcripts read per million maps (TPM). Spearman correlation coefficient (SCC) (Liesecke et al., 2018) between the three biological replicates of different samples is from 0.93 to 0.99, indicating the high quality of the replicates of each treatment (**Figure S2**).

Overall, *a total of ~81% genes were identified as expressed in at least one of the 54 samples.* At different stages of drought stress, the number of genes expressed in the root samples ranged from 41.4% (C16-25d-150) to 43.2% (C16-20d-0) in C16 and from 59.3% (C119-25d-150) to 61.1% (C119-25d-0) in C119 (**Figure S3** **A**). The expression levels of about 14% to 23% of the genes were very high (TPM ≥ 50) in root samples at different stages of drought stress (**Figure S3** **B**). The number of genes that had high (10 ≤ TPM ≥ 50), moderate (2 ≤ TPM ≥ 10), and low (0.1 ≤ TPM ≥ 2) expression levels was similar in all root samples at different stages of drought stress. In general, the proportion of *highly* expressed genes was slightly higher in C16 than in C119 (**Figure S3** **B**). Overall, the analyses showed sufficient coverage of transcriptomes during root response to drought stress in the two potato cultivars.

### Global comparison of transcriptomes revealed relations among root stages

**Figure 2:**
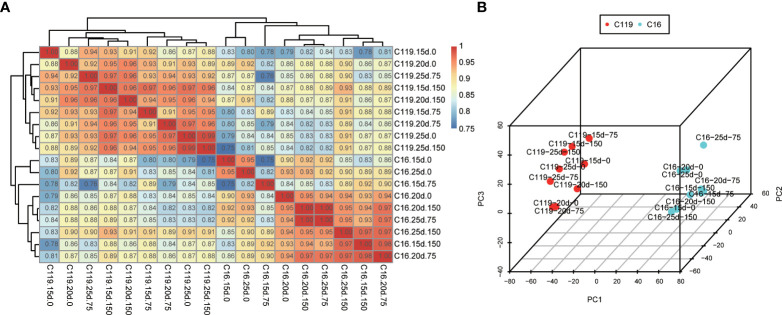
Correlations between transcriptomes of different stages of root response to drought stress in potato cultivars C16 and C119. **(A)** Spearman correlation coefficient analysis of RNA-seq data from root responses to drought stress. **(B)** Principal component (PC) analysis plot showing clustering of transcriptomes of root responses to drought stress. 15d, 20d and 25d represent the treatment days; 0, 75 and 150 represent the mannitol concentration.

To investigate global differences in transcriptome dynamics during root response to drought stress between C16 and C119 cultivars, hierarchical clustering and principal component analysis (PCA) were performed based on SCC analysis of the average TPM values of all genes expressed in at least one of the 54 root samples (**Figure 2**). Roots/stages with relatively high correlations were considered to have similar transcriptomes and associated functions. These analyses indicated that the root systems of the two cultivars were highly correlated at similar stages of drought stress. As expected, the root transcriptomes of each genotype were clustered together and showed significant differences in root responses drought stress stages (**Figures 2** **A, B**). Different stages of root drought stress response showed close correlations within the cultivars, indicating high similarity in transcriptional programs. Notably, the clustering of C119 and C16 under drought stress was significantly different, There is a large separation between the two varieties (**Figure 2** **B**). Overall, the results suggest there are large differences in the transcriptional programs of root responses to different stages of drought stress among cultivars. Furthermore, these large differences in transcriptional programs could determine cultivar-specific responses to drought stress and rooting depth.

### Gene sets differentially expressed between potato cultivars

**Figure 3:**
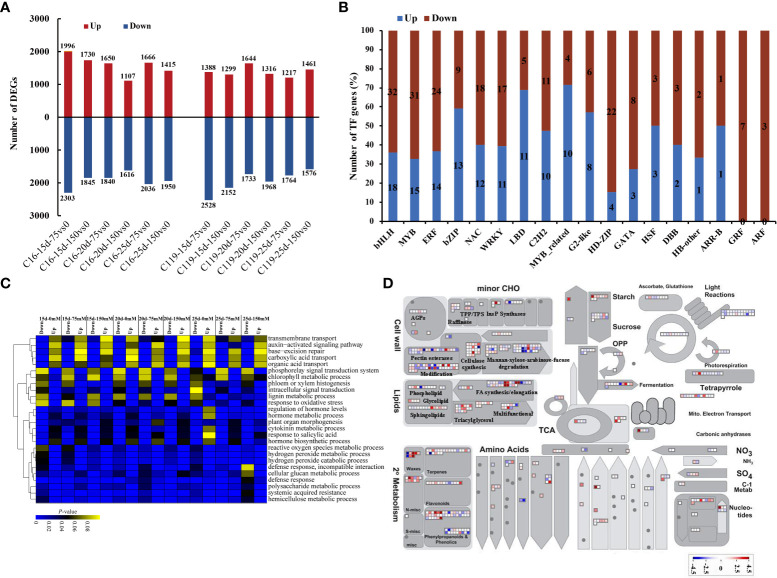
Differential gene expression in potato cultivars C119 and C16 at different stages of root development. **(A)** Number of up-regulated (red bars) and down-regulated (blue bars) differentially expressed genes (DEGs) at each stage of root development. **(B)** Number of genes from different transcription factor (TF) families up- or down-regulated in C119 during root responses at different stages of drought stress. **(C)** Enriched Gene Ontology terms (biological processes) for down- and up-regulated genes in C119 during root responses at different stages of drought stress. Lower color scale indicates significance (adjusted Pvalue). **(D)** Metabolic pathways with different expression profiles in C119 compared to those in C16 after 20 days of drought stress during root development. Differentially expressed genes (fold change ≥ 2, q-value ≤ 0.05) between C16 and C119 after 20 d of drought stress were loaded into MapMan to provide an overview. On the log2 scale, dark blue and dark red represent higher and lower expression, respectively, in C119 than in C16.

Gene sets were identified that showed significantly different expression between C16 and C119 at each stage of root response to drought stress. In C16, the expression of *9,564* genes were significantly increased*, and* of 11 590 genes was significantly decreased at different stages of root response to drought stress. *In C119, the expression of 8,325 genes was significantly increased, and of 11,721 genes was significantly decreased at different stages of root response to drought stress. In C16, the largest number of upregulated genes* (1,996) occurred at 15 days of drought stress, while in C119, the largest number of upregulated genes (1,644) occurred at 20 days of drought stress (**Figure 3** **A**). *Furthermore, in C16 and C119, we detected 2016 and 1766 differential genes, respectively, which were expressed under all drought stress treatments, suggesting that they had the ability to adapt to different drought stresses conditions (* **Figure S4** *).* Overall, *42 TF families* (transcription factors) in C119 showed differential expression than in C16, and these families had different functions during root response to different stages of drought stress (**Figure 3** **B**; **Table S2**). *For some TF families in C119, there are some genes with significantly high and low expression (* **Figure 3** **B** *).* For example, TF family members involved in cell proliferation and differentiation, such as ARF (auxin response factors) and GRF (growth regulatory factor), and those related to hormone signaling pathways, such as ARF and Aux/IAA (indole-3-acetic acid) and ARR-B (authentic typeB response regulator), and regulating polar development of lateral organs, such as HD-ZIP (homeodomain leucine zipper), showed significantly higher expression in C119 than in C16. Most TFs with lower expression in C119 than in C16 belonged to WRKY and Myb (myeloblastosis) -related families. **Figure S5** shows expression profiles of selected TF families in both cultivars during root response to different stages of drought stress.

Gene ontology enrichment (GO) of differentially expressed genes in C119 identified several biological processes that were unique to or related to root response at different stages of drought stress. Terms related to cell division, such as cell wall tissue, cell wall tissue or biogenesis, and plant cell wall tissue or biogenesis, were significantly enriched in relatively highly expressed genes (**Figure 3** **C**). The GO terms regulation of hormone level and hormone biosynthesis process were also highly enriched at different stages of drought stress. In particular, the GO terms phloem or xylem histogenesis and lignin metabolic processes were significantly enriched and relatively strongly expressed in upregulated genes at different stages of drought stress. In addition, the GO terms auxin-activated signaling pathway and base excretion repair were significantly enriched in relatively highly expressed down-regulated genes (**Figure 3** **C**).

To investigate the metabolic pathways responsible for the differences in rooting depth between C119 and C16 in response to drought stress, the MapMan tool was used to superimpose the expression profiles of differentially expressed genes (DEGs) between cultivars on the available metabolic pathways (Bolger et al., 2021). Differences in the activity of some metabolic pathways were observed during the 20-day drought stress period. There were significant differences in the transcriptional activity of genes related to starch biosynthesis between different cultivars. Genes involved in starch metabolism and ester synthesis were more active in C119 than in C16, suggesting that active cell division in deep-rooted C119 requires an increase in energy (**Figure 3** **D**). Genes involved in cell wall synthesis and modification also showed consistently higher expression in C119 than in C16 (**Figure 3** **D**). In addition, genes involved in DNA repair, DNA synthesis, regulation of transcription, RNA synthesis, protein synthesis, protein modification, and protein degradation and transport showed higher transcriptional activity in C119 than in C16 from day 15 to 25 under drought stress (**Figure S6**), indicating that deep roots were more active under drought stress. Overall, the data showed that there were large transcriptional differences between C16 and C119 at different stages of drought stress.

### Identification of gene coexpression modules of different potato cultivars

**Figure 4:**
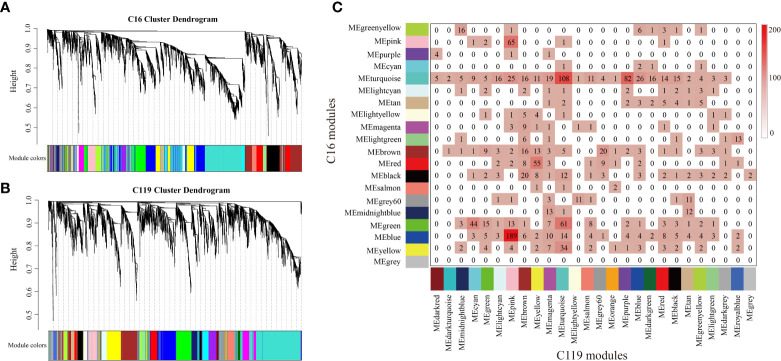
Coexpression networks in potato cultivars C16 and C119 during root development under drought stress. **(A, C)** hierarchical gene cluster tree (dendrogram) based on **(A)** C16 and **(B)** C119 coexpression network analysis. Each “leaf” (short vertical line) corresponds to a separate gene. Genes were clustered according to a diversity measure [1-TOM (topology model)]. The branches correspond to highly interconnected gene modules. The color bars under the dendrograms indicate module membership in **(A)** C16 and **(B)** C119. **(C)** Comparison of C16 and C119 modules based on cross-tabulation. Each C16 (row) and C119 (column) module is labeled by the corresponding module color. The numbers of genes for each intersection of the corresponding row and column modules are given. The color in the boxes represents numbers, according to the color legend on the right.

To investigate gene coexpression networks associated with responses of with shallow-rooted and deep-rooted cultivars to drought stress, the coexpression gene sets were determined by weighted gene coexpression network analysis (WGCNA). Genes with very low expression or low Spearman correlation coefficient (SCC) values were not included in the analysis to avoid inclusion of false edges in the network. Analysis of the differential gene regulatory networks of the two potato cultivars was performed separately. Several large subnetworks representing high coexpression between genes with similar expression profiles were identified as coexpression modules. Twenty modules (consisting of 89 to 1,198 genes) were identified in C16 (**Figures 4** **A** and **S7A**), whereas twenty-six modules (comprised of 101 to 1,873 genes) were identified in C119 (**Figures 4** **B** and **S7B**). Correlation analysis was used to determine the relationships between each coexpression module physiological indices [superoxide dismutase (SOD), peroxidase (POD), catalase (CAT), root vitality (RV), proline (Pro), soluble sugar (SS), root length (Len), root diameter (Diam), root volume (Vol), root tips (Tips), root forks (Forks)] of roots at different stages of drought stress were investigated (**Figures S7** **A, B**). In particular, 11 coexpression modules of C16 and 14 coexpression modules of C119 showed relatively high correlations (r ≥ 0.50) with roots responding to different stages of drought stress (**Figures S7** **A, B**). Many modules were correlated with more than one physiological index at different stages of drought stress. For example, the turquoise module of C16 was correlated with all physiological indices, with 0.72 being the highest correlation. In addition, with the intensification of drought stress, correlations between the red module and all physiological indicators tended to change from positive to negative (**Figure S7** **A**). In C119, the purple module was correlated with all physiological indices, with 0.73 the highest correlation. In addition, with the intensification of drought stress, correlations between the purple module and all physiological indicators tended to change from negative to positive, whereas those with the light cyan module tended to change from positive to negative (**Figure S7** **B**). Next, we studied the preservation of coexpression modules between C16 and C119 at different stages of drought stress *via* cross-tabulation approach. The modules with the most common genes are the turquoise and blue modules in C16, and the pink, turquoise and purple modules in C119, respectively (**Figure 4** **C**).

**Figure 5:**
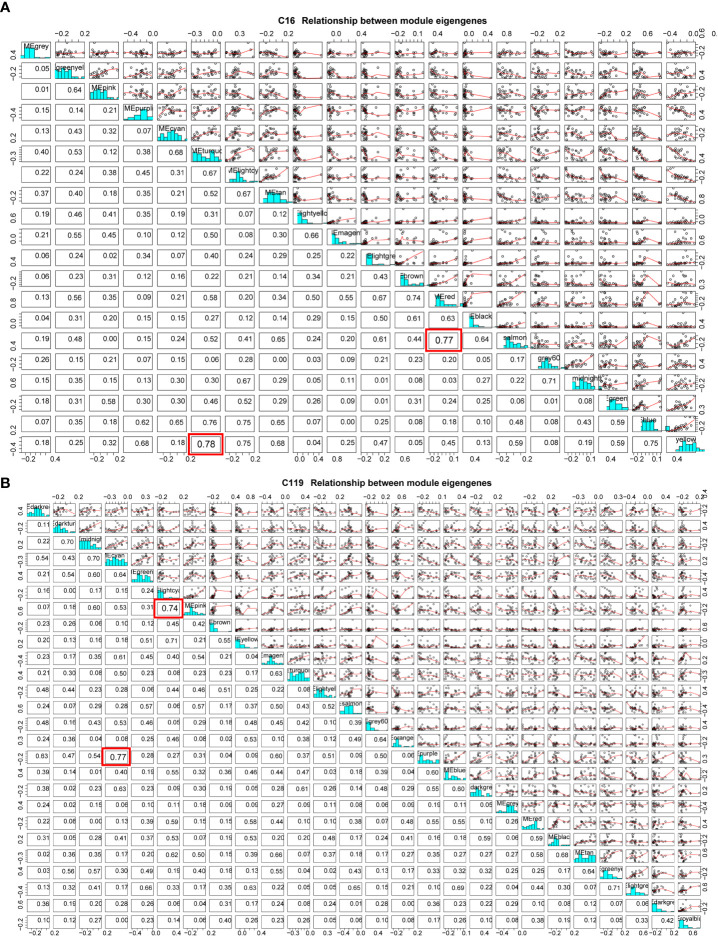
Module eigengene correlations between different modules in potato cultivars **(A)** C16 and **(B)** C119. Red box represents the two modules with the highest correlation.

Because of the enormous number of genes in a module, dimensionality reduction can be used to study gene modules by selecting representative genes in a module as module eigengenes (ME) (Langfelder and Horvath, 2007). Using a ME, which represents thousands of genes in a module for correlation analysis, the method can further clarify the relationships between gene modules and phenotypic traits at different stages of drought stress and identify the target gene module (Panahi and Hejazi, 2021). In the cluster analysis of MEs of all modules, some MEs were highly correlated (**Figure 5**). In C16, the correlation between MEs in yellow and turquoise modules reached 0.75 and that between MEs in salmon and red modules reached 0.74. As drought stress intensified, the correlations between gene expression patterns and stress resistance-related physiological and biochemical indicators gradually changed from positive to negative (**Figure 5** **A**). At C119, the correlation between MEs in cyan and purple modules reached 0.77 and that between MEs in light cyan and pink modules reached 0.74. With the exacerbating of drought stress, the correlations between gene expression patterns and stress resistance-related physiological and biochemical indicators in the purple module gradually changed from negative to extremely significant positive. Moreover, the correlations between gene expression patterns and target traits in the cyan and light cyan modules gradually changed from positive to extremely significant negative with the intensification of drought stress (**Figure 5** **B**).

### Transcriptional regulatory modules associated with rooting depth and root response to different stages of drought stress

**Figure 6:**
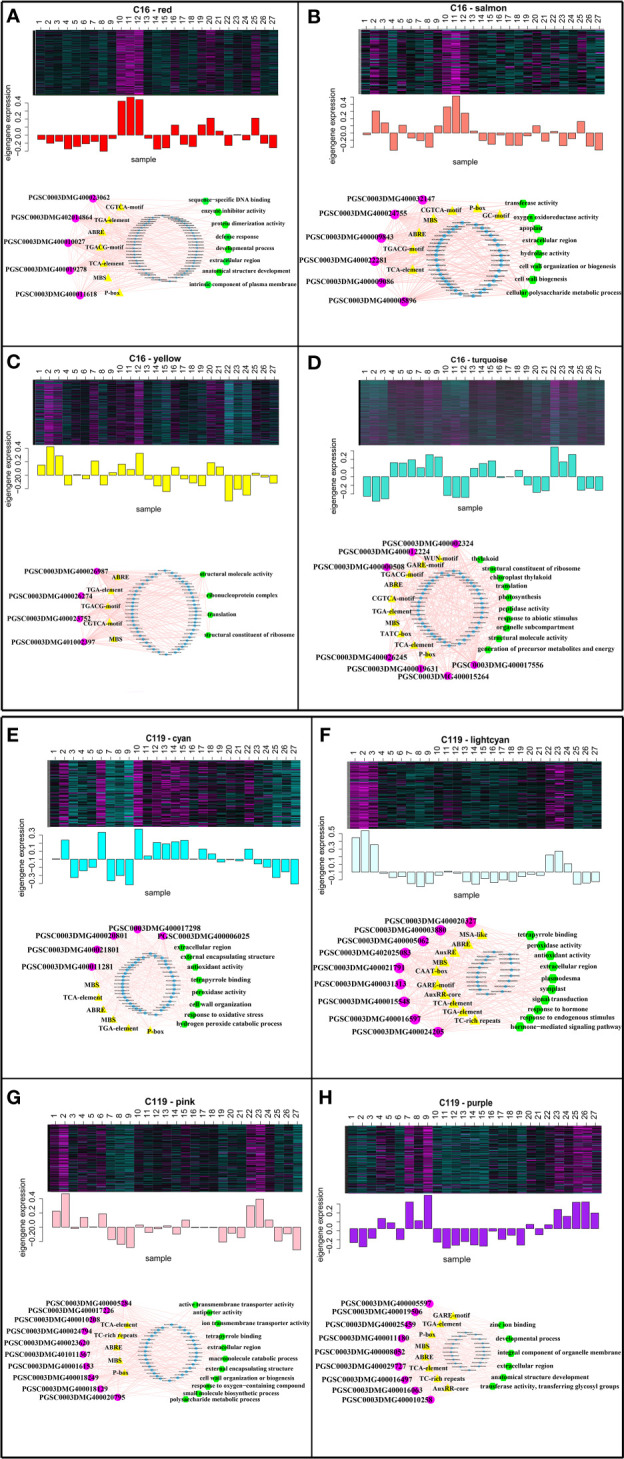
Expression profiles and transcriptional regulatory networks associated with modules in potato cultivars C16 and C119. Heatmaps show the expression profiles of all co-expressed genes in the modules (highlighted above). The bar graph (below the heatmap) shows the consistent expression pattern of the coexpressed genes in each module. The top-hub gene network is shown in circle below the bar graph. **(A–D)** Expression modules of C16, with networks of 5, 6, 4, and 7 hub genes, respectively. **(E-H)** Expression modules of C119, with networks of 5, 9, 10, and 9 hub genes, respectively. The names of the modules are indicated. The predicted transcriptional regulatory network [significantly enriched hub gene-binding sites along with the associated genes and enriched gene ontology (GO) terms] associated with the gene sets showing expression patterns at selected target gene modules are given. The significantly enriched cis-regulatory motifs (yellow triangles), GO terms (green hexagons) and other regulatory genes (blue circles) within the given set of genes. The hub genes are represented by magenta circles. Edges represent known interactions between the cis-regulatory motifs and hub genes.

To identify the hub genes in the eight modules, gene regulatory networks were visualized using Cytoscape software to identify the highly connected genes in the modules (Doncheva et al., 2019; Mousavian et al., 2021) (**Figure 6**). In a network, each node represents a gene, and lines connect the nodes, with genes at either end of a line usually involved in the same biological pathways (Mousavian et al., 2021). In C16, five nuclear genes were examined in the red module, six in the salmon module, seven in the turquoise module, and four in the yellow module. In C119, five nuclear genes were examined in the cyan module, nine in the light cyan module, ten in the pink module, and nine in the purple module. To obtain functional information on the nuclear genes, potato gene and NCBI databases (National Center for Biotechnology Information, https://www.ncbi.nlm.nih.gov/) were used to query relevant reports on the nuclear genes, and the TAIR database (https://www.arabidopsis.org/) was used to determine the functions of homologous genes of the nuclear genes in *Arabidopsis* (**Table S3**). The *PGSC0003DMG400032147* gene AT5G06720 homologous to C16 in *Arabidopsis* encodes a peroxidase with various functions in wound response, flower development, and syncytium formation (Shigeto et al., 2014). The homologous genes *AT5G40950* of *PGSC0003DMG400019631* and *AT5G56670* of *PGSC0003DMG401002397* in *Arabidopsis* may encode ribosomal proteins and participate in protein synthesis (Wang et al., 2008; Bach-Pages et al., 2020). The homologous gene of *PGSC0003DMG400009843* in *Arabidopsis AT5G67200* is involved in receptor protein kinase synthesis and regulates plant growth and development (ten Hove et al., 2011). In C119, the gene *AT3G61060* homologous to *PGSC0003DMG400010258* in *Arabidopsis* is involved in the formation of plant root phloem, and roots play a central role in plant growth, development, and stress response (Hanada et al., 2011). The homologous gene *AT3G01190* of *PGSC0003DMG400021801* in *Arabidopsis* has synergistic or antagonistic effects with plant hormones by regulating reactive oxygen species and redox signalling to elicit protective responses of plants to biological and abiotic stresses (Welinder et al., 2002). The gene *AT5G05340* homologous to *PGSC0003DMG400005062* in *Arabidopsis* regulates the formation of plant root xylem by encoding a protein involved in lignin biosynthesis (Fernández–Pérez et al., 2015). The gene *AT3G09790* homologous to *PGSC0003DMG400021791* in *Arabidopsis* is associated with the specificity of ubiquitination and may promote the transfer of ubiquitin to appropriate targets and regulate cell activities (Bachmair et al., 2001). Overall, the analysis identified transcriptional modules as the key regulatory network, and identified the hub genes of shallow-rooted and deep-rooted potato varieties in response to drought stress.

Hub gene binding sites (significantly enriched motifs) were identified in the roots of C16 and C119 in response to different stages of drought stress. This resulted in prediction of transcriptional modules linking the enriched regulatory motifs with the hub genes involved in different stages of drought stress and their association with specific GO terms represented significantly in the target genes. The transcriptional module in C16 (red, salmon, turquoise, and yellow modules) included significantly enriched GGTCA-motifs, GARE-motif, GC-motifs, ABRE, MBS, P-box, TATC-box, TCA-elements, TGA-elements, and TGACG-motifs. These motifs are mainly a gibberellin-responsive element, a MeJA-responsive element, an abscisic acid-responsive element, an auxin-responsive element, a salicylic acid-responsive element, and an MYB-binding site involved in dryness induction. The transcriptional modules included target genes involved in sequence-specific DNA binding, extracellular region, anatomical structure development, defense response and developmental process(red module); transferase activity, oxygen oxidoreductase activity, extracellular region, hydrolase activity and cell wall organization (salmon module); structural molecule activity, ribonucleoprotein complex, translation and structural constituent of ribosome (yellow module); structural constituent of ribosome, translation, response to abiotic stimulus, organelle subcompartment and structural molecule activity(turquoise module) (**Figures 6** **A–D**). Many of these components were similar to the transcriptional module in C119 (cyan, light cyan, pink, and purple modules), with some specific components including motifs, AuxRE, AuxRRcore, MSA-like, and TC-rich. Some of these regulatory motifs are known to be associated with elements involved in cell cycle regulation, elements involved in defense and stress response, and regulatory elements involved in auxin response. The transcriptional modules included target genes involved in extracellular region, external encapsulating structure, antioxidant activity, peroxidase activity, response to oxidative stress and cell wall organization (cyan module); peroxidase activity, antioxidant activity, extracellular region, signal transduction and response to endogenous stimulus (lightcyan module); active transmembrane transporter activity, tetrapyrrole binding, extracellular region, cell wall organization and response to oxygen−containing compound (pink module); zinc ion binding, developmental process, integral component of organelle membrane, extracellular region and transferase activity (purple module) (**Figures 6** **E–H**). Overall, these analyses identified key regulators of root response to drought stress and suggested that root response to drought stress processes are regulated differentially in the two potato cultivars.

### Reverse-transcription quantitative PCR verification of core genes

**Figure 7:**
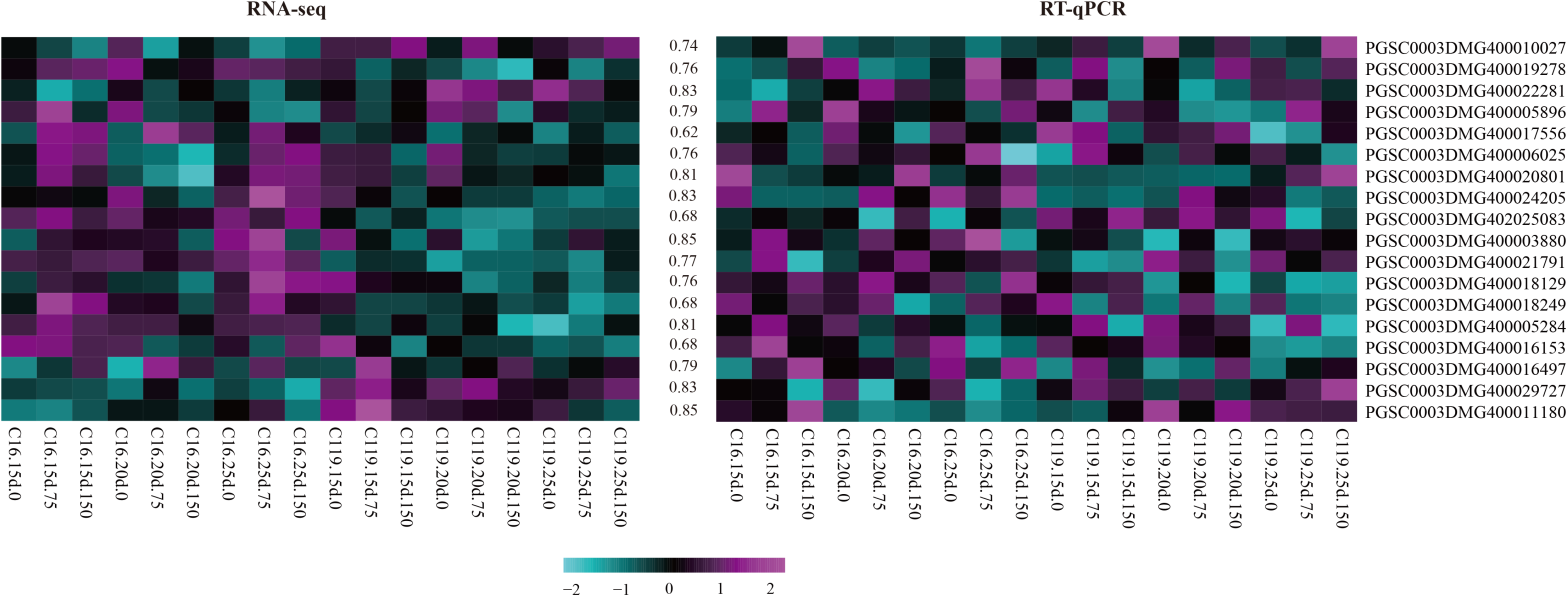
Heat maps of correlations between expression profiles of selected genes (highlighted on the right) obtained from RNA-sequencing (RNA-seq) and quantitative reverse-transcription PCR (RT-qPCR). The lower color scale represents *Z*-scores. The values between the two heatmaps represent the correlation coefficients between the expression profiles obtained from RNA-sequencing and RT-qPCR analysis of each gene.

Fifty-five core genes potentially associated with drought stress were selected from the eight gene modules with the highest correlations (**Table S3**), and 18 candidate genes involved in different processes were randomly selected. These genes were selected as candidate genes to determine the mechanisms regulating potato rooting depth and root response to drought stress. Gene expression was verified by fluorescent quantitative reverse transcription PCR (RT-qPCR). The backup RNA used for transcriptome sequencing was reverse transcribed, and the responses of the 18 genes to drought stress in C16 and C119 were detected by RT-qPCR. The changes in expression were generally consistent with transcriptome results (**Figure 7**), further demonstrating the reliability of the transcriptome sequencing results and confirming the response of the major genes to drought stress.

## Discussion

The molecular mechanisms underlying differences in rooting depth and root response to drought stress are poorly understood in potato. In this study, RNA-seq was used to determine transcriptome dynamics in two potato cultivars with different rooting depths (shallow vs. deep) at different stages of drought stress. The molecular mechanisms underlying root responses to drought stress and differences in rooting depth were then investigated. RNA-seq investigation allowed the discovery of new genes and their expression profiles. The expression data of the three root responses to drought stress phases showed high reproducibility in both potato cultivars and clear separation between cultivars in the PCA (principal component analysis) diagram, suggesting that potato cultivars may have different mechanisms for responding to drought stress. The dataset comprehensively describes the transcriptional activity of potato roots at different stages of drought stress. In coexpression networks and transcriptional modules, several coregulatory and specific transcriptional programs within and between cultivars have been linked to rooting depth and root response to drought stress.

The differential distribution of resources during root formation determines whether roots are deep or shallow in different plant species (Zhou et al., 2020b). In addition, greater root depth is associated with increases in other traits, including the number of lateral roots, stem thickness, and root strength (Gala et al., 2021). Common genetic components are thought to regulate root number and other organs *via* effects on cell cycle and developmental duration (Linkies et al., 2010). A prolonged phase of cell division that increases the number of cells in root hairs or lateral roots is thought to be responsible for root depth (Fernandez et al., 2020). Root length of the two potato cultivars increased after 20 days of drought stress, suggesting that this period represents a transition between cell division and maturation. The difference in the rate of increase in root length suggests that there were differences in the number of cell divisions between the two potato cultivars. Gene ontology enrichment analysis showed that C119 has a prolonged cell division cycle and relatively high expression of genes involved in cell division and cell cycle. In maize, a prolonged root growth period is associated with relatively high gluconeogenesis, which plays a key role in determining root length (Yu et al., 2021). The increased cell number in C119 could lead to increased accumulation of storage substances and proteins and stimulation of cell growth and proliferation. Meristem cell number and root length were also positively correlated (Di Mambro et al., 2019). In C119, the enriched GO terms mainly included peroxidase activity, cellular response to hormonal stimuli, and integral component of peroxisomal membrane, suggesting a relatively high cell division ability and an important role of peroxide signalling in determining potato root responses to drought stress. A crucial role of peroxidase in determining root responses to drought stress was also found in a previous study (Su et al., 2018). Moreover, relatively the high transcriptional activity of several genes related to the metabolic process of reactive oxygen species, signal transduction, response to oxygenated compounds, transferase activity, and glycosyl group transfer was associated with drought resistance in the deep-rooted potato cultivar. In previous studies, we found that C16 and C119 have significant differences in phytohormone signal transduction pathways, which is consistent with our previous findings (Qin et al., 2022). Thus, a prolonged cell division period that increases the number of cells could explain the deep rooting and strong drought resistance of C119.

Many TF families have been associated with root development (Yamada et al., 2020; Zhang et al., 2021) but few TFs have been associated with root length determination and drought resistance. In the data set of this study, about 60% of TFs were differentially expressed between C16 and C119, suggesting differences in transcriptional regulatory networks between potato cultivars. Among the differentially expressed TF-coding genes, TF families with known functions were represented; however, the exact functions of most of these genes remain unknown. The role of some members of TF families, such as ARF, bZIP, MYB-related, and WRKY, which exhibited differential regulation among potato cultivars, are well known in root responses to drought stress (Jiang et al., 2017; Joo et al., 2021; Wei et al., 2021; Yang et al., 2021). Differential regulation of the same gene family members in different potato cultivars may result in different regulatory networks that can determine cultivar-specific rooting depth and root response to drought stress. To better understand root responses to drought stress, coexpression networks were analyzed, and unique gene sets and modules were determined that contained a significant number of hub genes associated with responses to different stages of drought stress in both cultivars. Hub genes may serve as regulatory components to coordinate the activities of co-expressed genes within a module. The GO enrichment analysis of the modules highlighted the role of different biological processes and pathways in root response to drought stress and suggested that different regulatory pathways control root depth in response to drought stress.

In *Arabidopsis thaliana*, root regulatory gene circuits in response to drought stress have been elucidated based on comprehensive transcriptome mapping of roots (Rasheed et al., 2016; Bashir et al., 2018). Transcriptional module discovery can identify coexpression networks that control biological processes associated with root development (Du et al., 2020; Wang et al., 2021b). Therefore, we generated transcriptional modules of nuclear genes for potato root development and co-expressed target genes in C16 and C119 that could determine the differences in root development and rooting depth between the two potato cultivars. Analysis of potential binding motifs on the hub genes revealed that despite the extensive overlap of motifs on the hub genes of C16 and C119, several components exhibit cultivar specificity, which determines the unique transcriptional modules of the two potato cultivars.

This study has shown that transcriptional modules construction and coexpression network analysis are powerful tools for understanding the molecular mechanisms underlying agronomic traits, such as root development and root response to drought stress. However, further functional studies on individual members of the network are needed in order to fully understand the coexpression network.

## Materials and methods

### Plant Material

Two potato genotypes, C16 (CIP 397077.16, shallow rooted) and C119 (CIP 398098.119, deep-rooted), were provided by the International Potato Research Center (Peru). Both genotypes had the same growth cycle but different root structures and drought resistance. The Key Laboratory of Crop Genetic Improvement and Germplasm Innovation at Gansu Agricultural University provided tissue cultured seedlings of C16 and C119 (**Table 1**). Stem sections of potato test-tube plantlets were transferred to paper boats floating on liquid MS (Murashige and Skoog) medium containing 0 mM (control) or 75 mM or 150 mM mannitol (drought stress treatments) (Hamooh et al., 2021; Sattar et al., 2021). After 15, 20, and 25 days of growth, the paper boats containing the tube seedlings were carefully removed. There were three biological replicates for each treatment. After treatment, the roots of the seedlings were removed, immediately frozen in liquid nitrogen extraction, and then stored in the refrigerator at −80°C. Half of each stored sample was used for RNA for subsequent transcriptome sequencing. The other half was used to determine physiological and biochemical indicators of stress resistance.

**Table 1:** Summary of the plant material used in this study.

Variety	CIP Number	Maternal Parent	Paternal Parent
C 16	CIP397077.16	392025.7 (=LR93.221)	392820.1 (=C93.154)
C 119	CIP398098.119	393371.58	392639.31

### Illumina sequencing, read mapping, and differential gene expression analyses

For RNA-seq, total RNA extraction and library preparation for each sample were performed as previously described (Garg et al., 2016). All 54 libraries (1 sample with three biological replicates) were sequenced on an Illumina platform (Zhang et al., 2014), Beijing Genomics Institute, Shenzhen, Guangdong, China) to generate double-end sequence reads of 150 nucleotides. The NGS QC toolkit (Patel and Jain, 2012), as previously described (Garg et al., 2016), was used to evaluate the various quality parameters of the original sequence data and philtre high-quality reads. The filtered high-quality reads were mapped to the potato genome (Felcher et al., 2012) using TopHat (v2.0.0, Centre for Bioinformatics and Computational Biology at the University of Maryland, College Park, MD, USA) with default parameters. The mapped output was processed through Cufflinks (v2.0.2) to obtain TPM (transcripts per million) for all potato genes in each sample. Correlations between biological replicates were determined by calculating the SCC (Spearman correlation coefficient). The corrplot and scatterplot3d utilities of the R software package (version 4.1.1) were used for hierarchical clustering and PCA, respectively. Differential expression between different samples was measured using the DESeq2 of the R package. After correction for false discovery rate (Qvalue), genes with at least a *twofold* difference and a corrected P-value < 0.05 were considered significantly differentially expressed. For a given set of genes, the Z-score of a line was determined, and the heatmap2 of the R package was used to generate the heatmap.

### Gene ontology and pathway enrichment analysis

Gene ontology enrichment analysis of DEG sets was performed using the AgriGo online tool (http://systemsbiology.cau.edu.cn/agriGOv2/index.php) (Tian et al., 2017; Liu et al., 2018). The enrichment P-value of each representative GO term was calculated and then corrected by the Benjamini–Hochberg error correction method. The significantly enriched GO terms had a corrected (after adjusting for false discovery rate) P-value ≤ 0.05. Pathway enrichment across different genomes was analyzed using MapMan (v3.5.1R2) classification for optimal Arabidopsis (TAIR10, https://www.arabidopsis.org/) homologues.

### Reverse-transcription quantitative PCR analysis

Results of RNA-seq were verified by RT-qPCR experiment. Reverse-transcription PCR analysis was performed as previously described (Garg et al., 2016). First-strand cDNA was synthesized using a PrimeScript RT kit (Takara Bio Inc., Shiga, Japan). The gene-specific primers designed with Prime3Plus (http://primer3plus.com/cgi-bin/dev/primer3plus.cgi) are listed in **Table S4**. The internal reference gene was Actin I (Kühn and Mannherz, 2017). The qPCR was performed on a Quant Studio 5 (Life Technologies Holdings Pte Ltd., Singapore) using SYBR Premix Ex Taq II (Tli RNaseH Plus; Takara Bio Inc.). PCR analysis was performed using three biological replicates of each sample and at least three technical replicates of each biological replicate.

### Determination of physiological and biochemical indicators

Physiological and biochemical indices (superoxide dismutase (SOD), peroxidase (POD), catalase (CAT), root vitality (RV), proline (pro), soluble sugar (SS)) were determined using a kit (elisa., Shanghai, China). Root length (Len), root diameter (Diam), root volume (Vol), root tips (Tips), and root forks (Forks) were measured using a root scanner (EPSON EXPRESSION 10000XL., Suwa, Japan). Each index was establishe with three biological replicates and three technical replicates.

### Coexpression network analysis for construction of modules

The WGCNA package was used for coexpression network analysis (Massa et al., 2011; Childs et al., 2011; Jain et al., 2017; Ma et al., 2021). Based on log2 (1+TPM) values, the paired SCC matrix between all gene pairs was generated and converted to an adjacency matrix (connection strength matrix) using the following formula: Strength of connection (adjacency value) = | (1 + correlation)*/*2| β, where β represents the soft threshold of the correlation matrix that gives greater weight to the strongest correlation while maintaining gene–gene connectivity. A β- value of 10 was chosen according to the scale-free topological criteria described by Yang and Horvath (Langfelder and Horvath, 2008; Zhao et al., 2021). The adjacency matrix was converted to a topological overlap matrix (TO) by a TOM similarity algorithm, and genes were hierarchically clustered based on similarity. A dynamic tree pruning algorithm was used to prune the hierarchical cluster tree, and modules were defined after decomposing and combining branches to achieve a stable number of clusters (Langfelder and Horvath, 2008). For each module, the summary contour (characteristic module gene, ME) was calculated by PCA. In addition, modules with values (the average TO of all genes in a given module) that were higher than the TO values of modules consisting of randomly selected genes were selected. *The Homer software was used for motif enrichment analysis (* Zhu et al., 2021 *).* Cytoscape and Agrigo were used for GO enrichment analysis of each module.

## Conclusions

In summary, RNA-seq data from two potato cultivars with different rooting depths (shallow and deep) provide a robust resource for studying potato root biology, including root development and root response to drought stress. Gene sets expressed at specific stages were identified, enriched biological processes and pathways were determined, and co-expressed gene sets were defined with high temporal resolution. In addition, genes coexpression networks that play a role in the response of deep and shallow roots to different stages of drought stress were identified, and nodal genes that regulate stage-specific root responses to drought stress were investigated. A prolonged period of cell division and an increase in peroxidase activity were primarily responsible for deep rooting and strong drought resistance in potato C119. Correlations between TFs with known functions and genes related to rooting depth and drought stress response identified 18 potential target genes that could determine rooting depth and root response to drought stress. Overall, this study demonstrates that transcriptional profiling and inferred coexpression networks, together with plant physiology approaches, can help identify the most promising candidate genes and determine the precise role in root response to drought stress. The comprehensive information presented in this study can be an important resource to improve our understanding of root responses to drought stress, especially the differences between deep-rooted and shallow-rooted potatoes. These new findings greatly improve our understanding of the genetic and molecular basis of drought resistance in two different potato genotypes and provide important clues for molecular breeding of drought-resistant cultivars.

## Data availability statement

The datasets presented in this study can be found in online repositories: https://www.ncbi.nlm.nih.gov/bioproject/?term=PRJNA851475.

## Author contributions

Conceptualization, TQ; methodology, TQ, AK; investigation, TQ, CS; data curation, TQ, CS, and JB; writing—original draft preparation, TQ; writing—review and editing, CS, YW. DR, AK, PY, ZB, YL, and JB; All authors have read and agreed to the published version of the manuscript.

## Funding

This research was funded by the National Natural Science Foundation of China (Grant No. 32060502 and 31960442), Agriculture Potato Research System of MOF and MARA (CARS-09-P10), the Gansu Science and Technology fund (Grant No. 22JR5RA835, 22JR5RA833, 20YF8WA137, 21JR7RA804 and 19ZD2WA002-02).

## Conflict of interest

The authors declare that the research was conducted in the absence of any commercial or financial relationships that could be construed as a potential conflict of interest.

## Publisher’s note

All claims expressed in this article are solely those of the authors and do not necessarily represent those of their affiliated organizations, or those of the publisher, the editors and the reviewers. Any product that may be evaluated in this article, or claim that may be made by its manufacturer, is not guaranteed or endorsed by the publisher.
