# Supplementary material for: Global transcriptome and coexpression network analyses reveal cultivar-specific molecular signatures associated with different rooting depth responses to drought stress in potato
Source: Front Plant Sci. 2022 Oct 19;13:1007866. doi: 10.3389/fpls.2022.1007866 (PMC9629812; doi:10.3389/fpls.2022.1007866)
Supplement: Supplementary file 1 [file DataSheet_1.zip › legends.docx]

**Figure S1.** Phenotypes of C16 and C119 under different drought stress.

**Figure S2.** Heatmaps showing correlation between transcriptomes of three biological replicates of each tissue sample from C16 and C119. Spearman correlation coefficient (SCC) among the replicates of C16 (a) and C119 (b) tissues is shown.

**Figure S3.** Gene expression in C16 and C119. Total number of genes expressed (a) and fraction of genes expressed at different expression levels (based on FPKM) (b) in different growth stages and different drought stress in C16 and C119 are shown in the bar graphs.

**Figure S4.** UpSet diagrams showing the numbers of DEGs in each variety (a: C16, b: C119). Vertical bars of upper plot show number of intersecting differential gene (DEGs) among different drought stress treatments, denoted by connected black circles below the histogram. Orange bars and circles represent DEGs that overlap among six drought stress treatments, horizontal bars show DEGs set size.

**Figure S5.** Heatmaps showing the TPM value expression profiles of members of selected TF families with preferential expression in C16 or C119 cultivar during root growth stages and drought stress. The color scale on the left represents Z-score.

**Figure S6.** Cellular response pathways showing differential expression between C16 and C119 at different growth stages and different drought stress. Differentially expressed genes (fold change ≥ 2, q-value ≤ 0.05) between C16 and C119 at each stage were loaded into MapMan to generate the overview. On the log2 scale, dark blue color represents higher expression in C16, while dark red color signifies higher expression in C119.

**Figure S7.** Association analysis of gene coexpression network modules with physiological and biochemical traits in C16 (a) and C119 (b). Each module is labeled with the corresponding module color. The horizontal axis shows the different features, and the vertical axis represents the eigenvector of each module. Pearson correlation coefficient of each module with different stages are given and colored according to the score.

**Table S1.** Summary of read data generated, quality control and mapping on the potato genome for different samples for C16 and C119 cultivars.

**Table S2.** List of genes associated used for construction of gene regulatory networks in the potato cultivars.

**Table S3**. Functional annotations of hub genes in different modules.

**Table S4.** Primers used for qRT-PCR.
