# Supplementary figures and images for: Global transcriptome and coexpression network analyses reveal cultivar-specific molecular signatures associated with different rooting depth responses to drought stress in potato
Source: Front Plant Sci. 2022 Oct 19;13:1007866. doi: 10.3389/fpls.2022.1007866 (PMC9629812; doi:10.3389/fpls.2022.1007866)

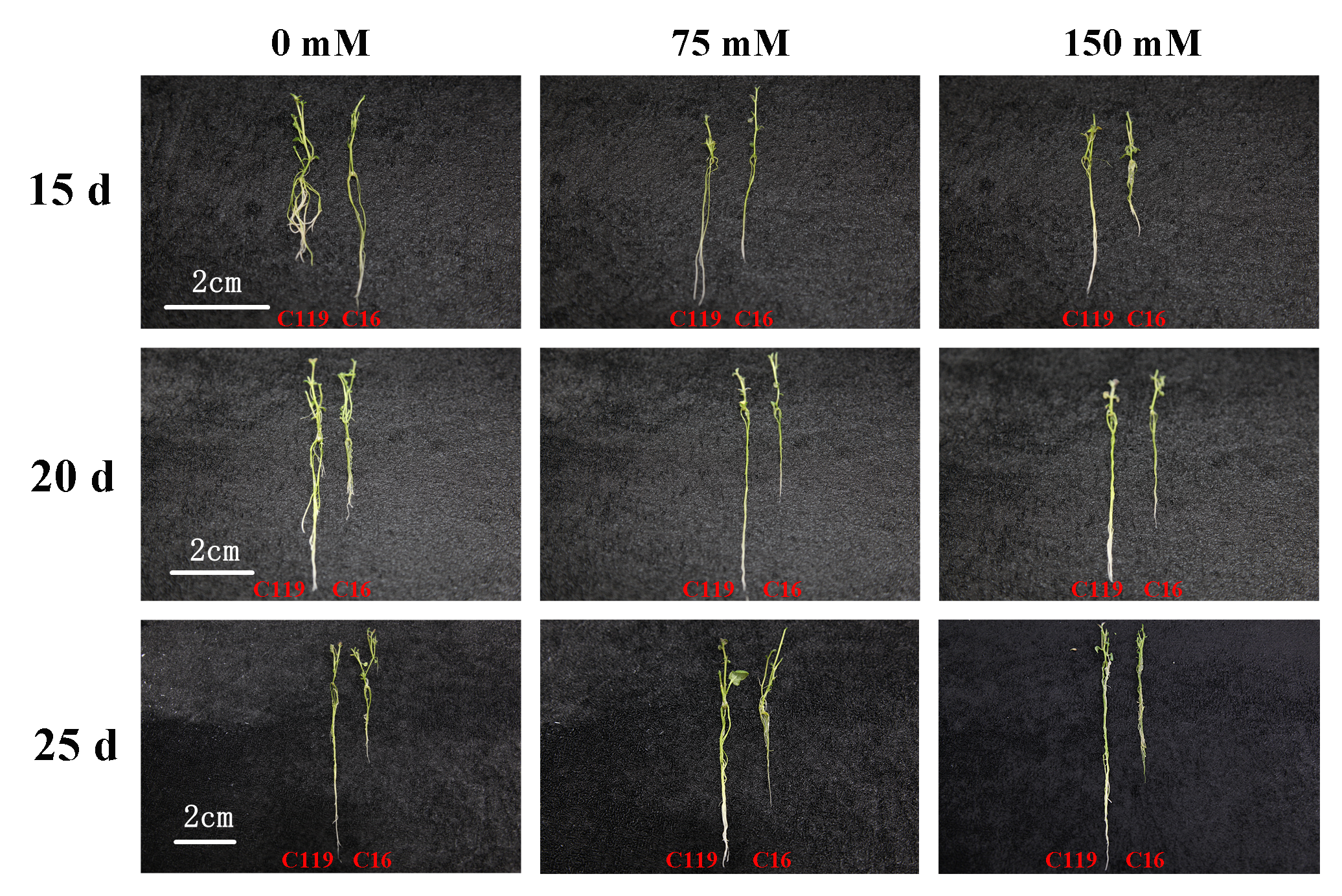

Supplement: Supplementary file 1 [file DataSheet_1.zip › Figure S1.tif]

A

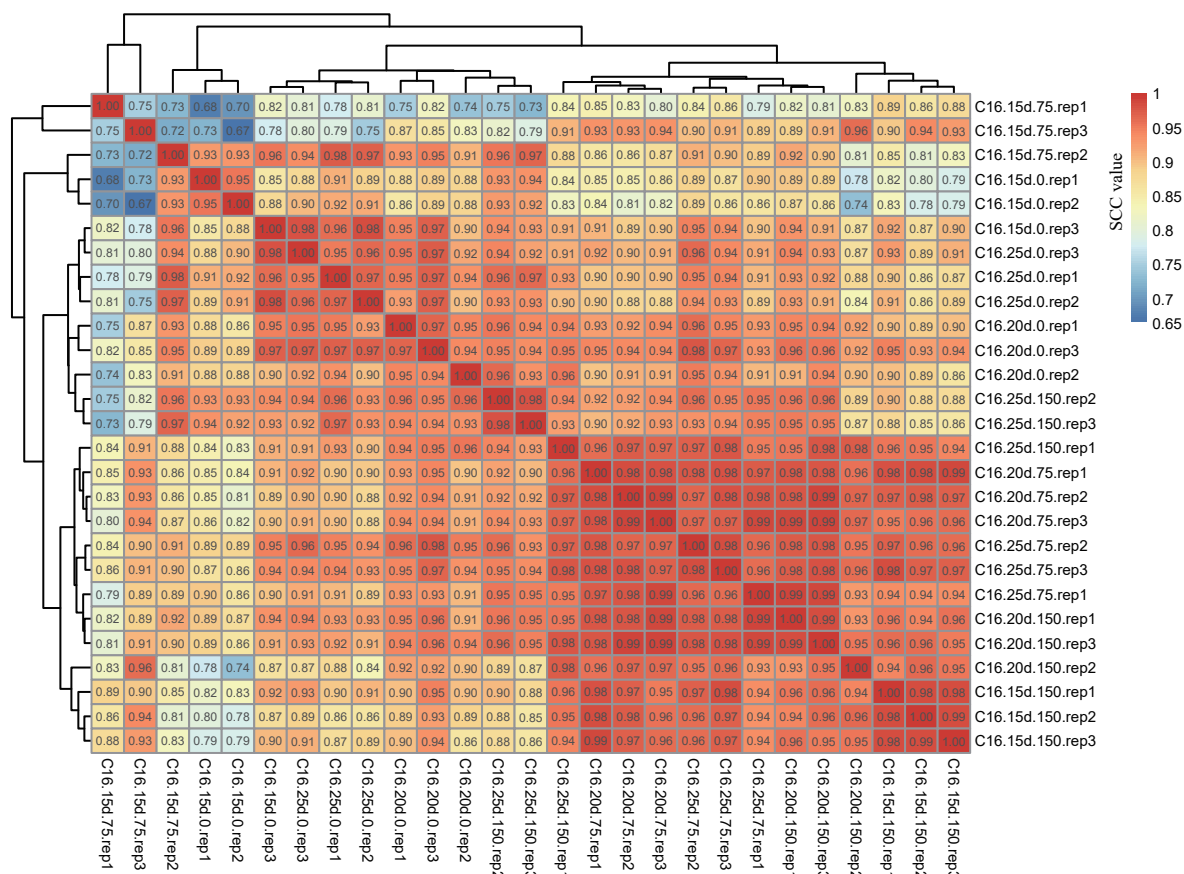

B

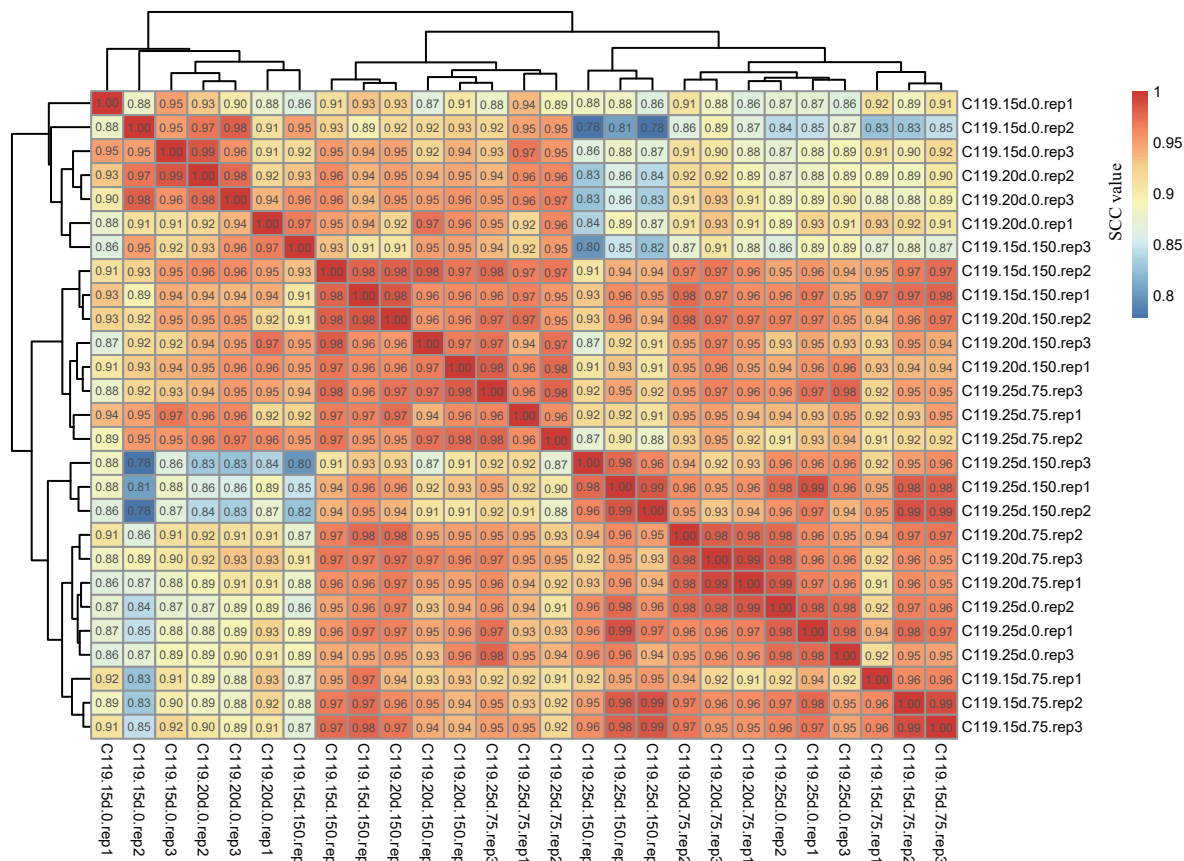

Supplement: Supplementary file 1 [file DataSheet_1.zip › Figure S2.pdf]

A

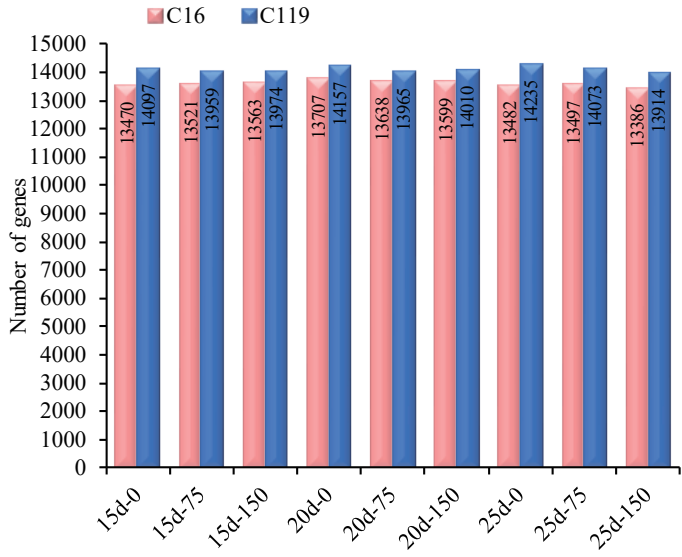

B

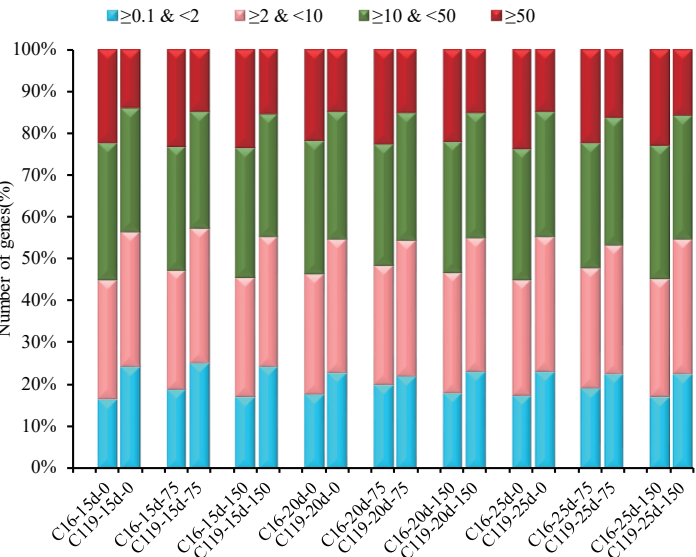

Supplement: Supplementary file 1 [file DataSheet_1.zip › Figure S3.pdf]

A

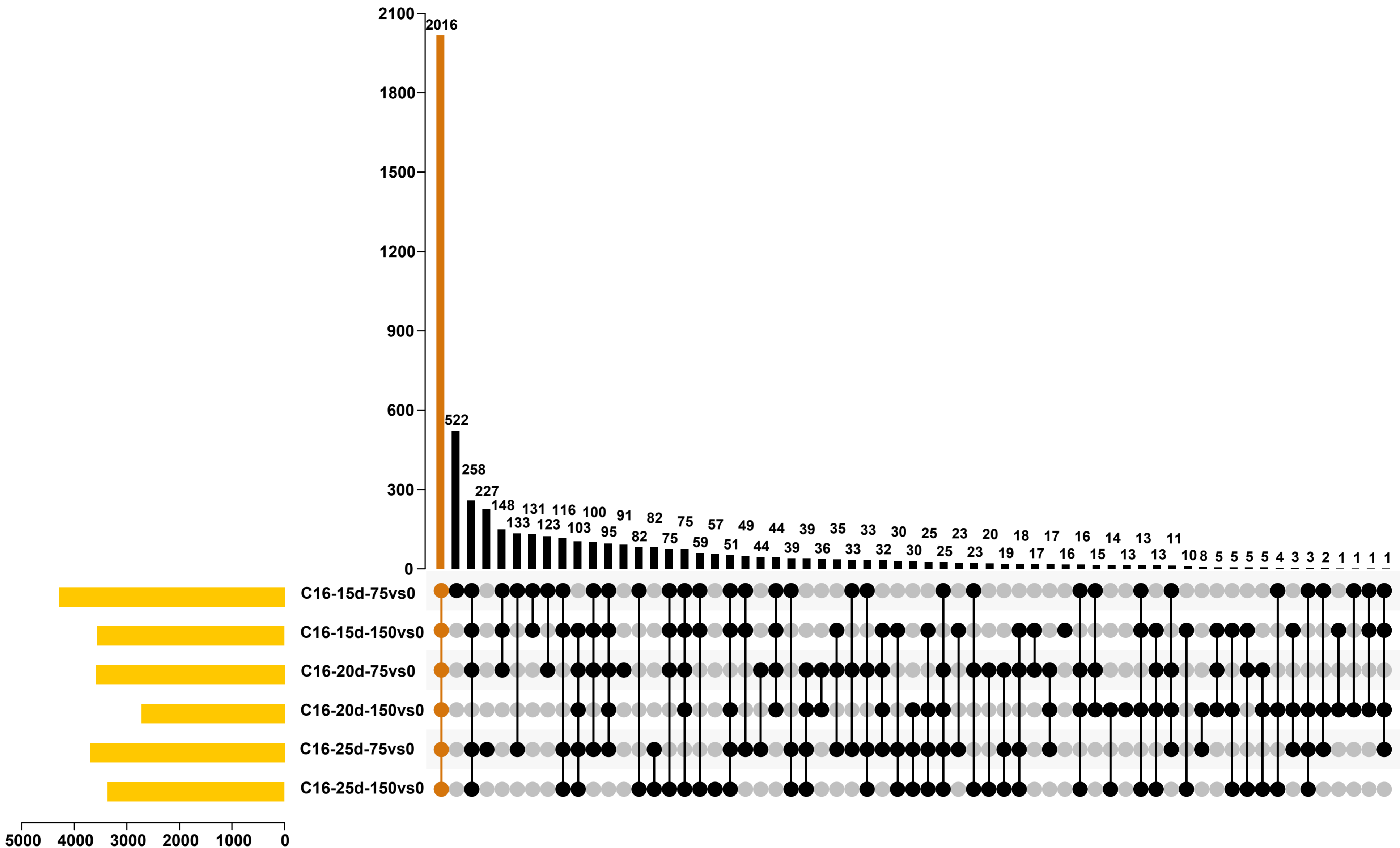

B

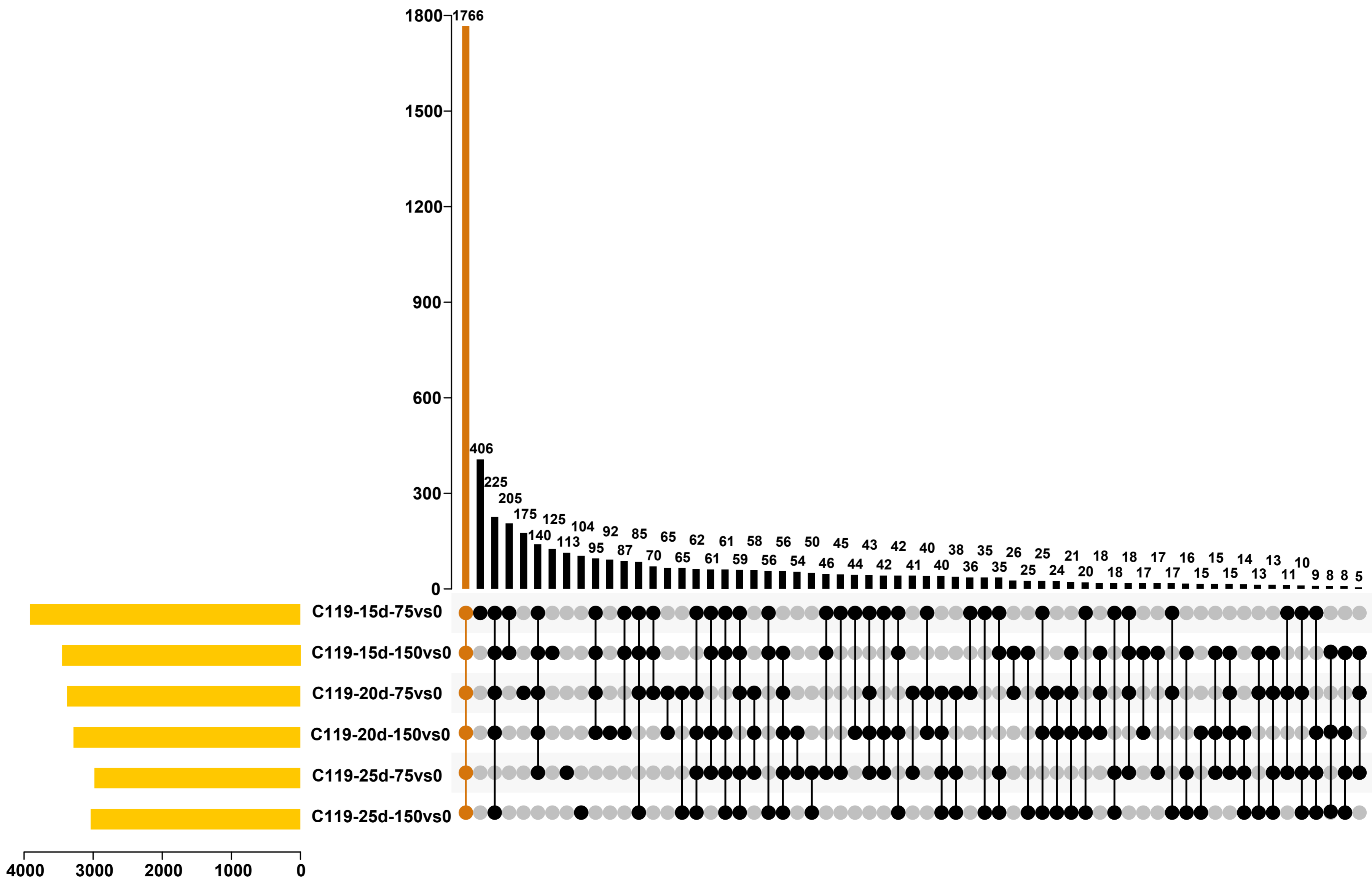

Supplement: Supplementary file 1 [file DataSheet_1.zip › Figure S4.pdf]

A

## C16 Module-trait relationships

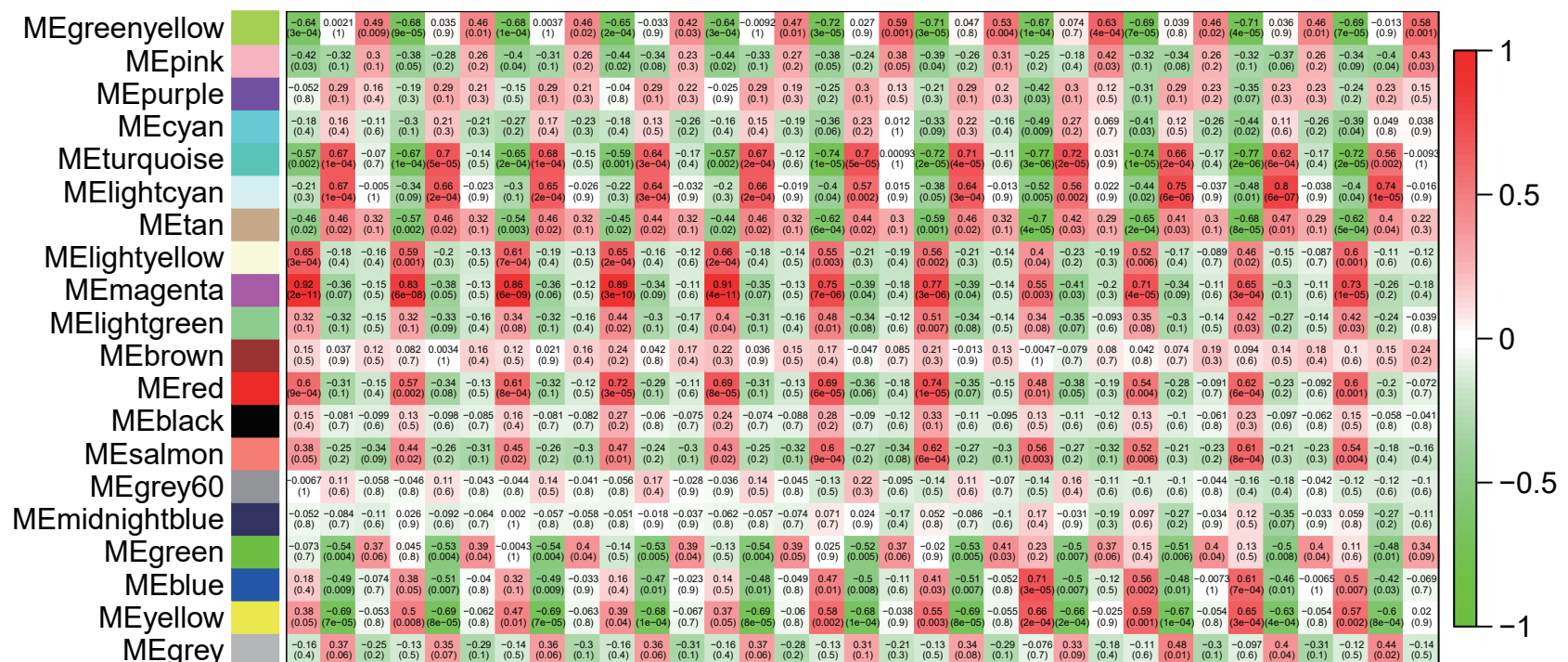

B

## C119 Module-trait relationships

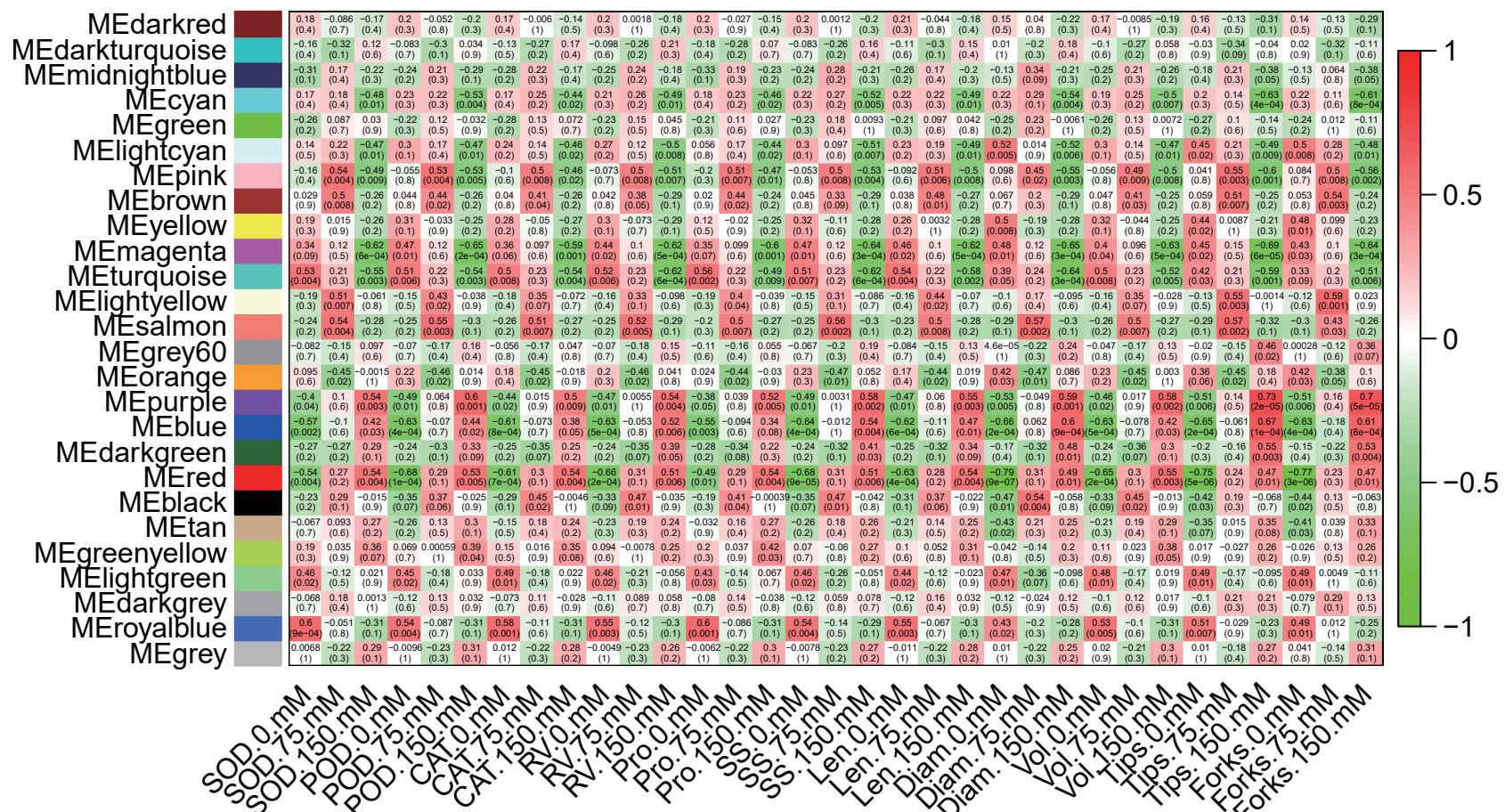

Supplement: Supplementary file 1 [file DataSheet_1.zip › Figure S7.pdf]
