# Supplementary material for: Global transcriptome and coexpression network analyses reveal cultivar-specific molecular signatures associated with different rooting depth responses to drought stress in potato
Source: Front Plant Sci. 2022 Oct 19;13:1007866. doi: 10.3389/fpls.2022.1007866 (PMC9629812; doi:10.3389/fpls.2022.1007866)
Supplement: Supplementary file 1 [file Figure S5.pdf › Figure S5.pdf]

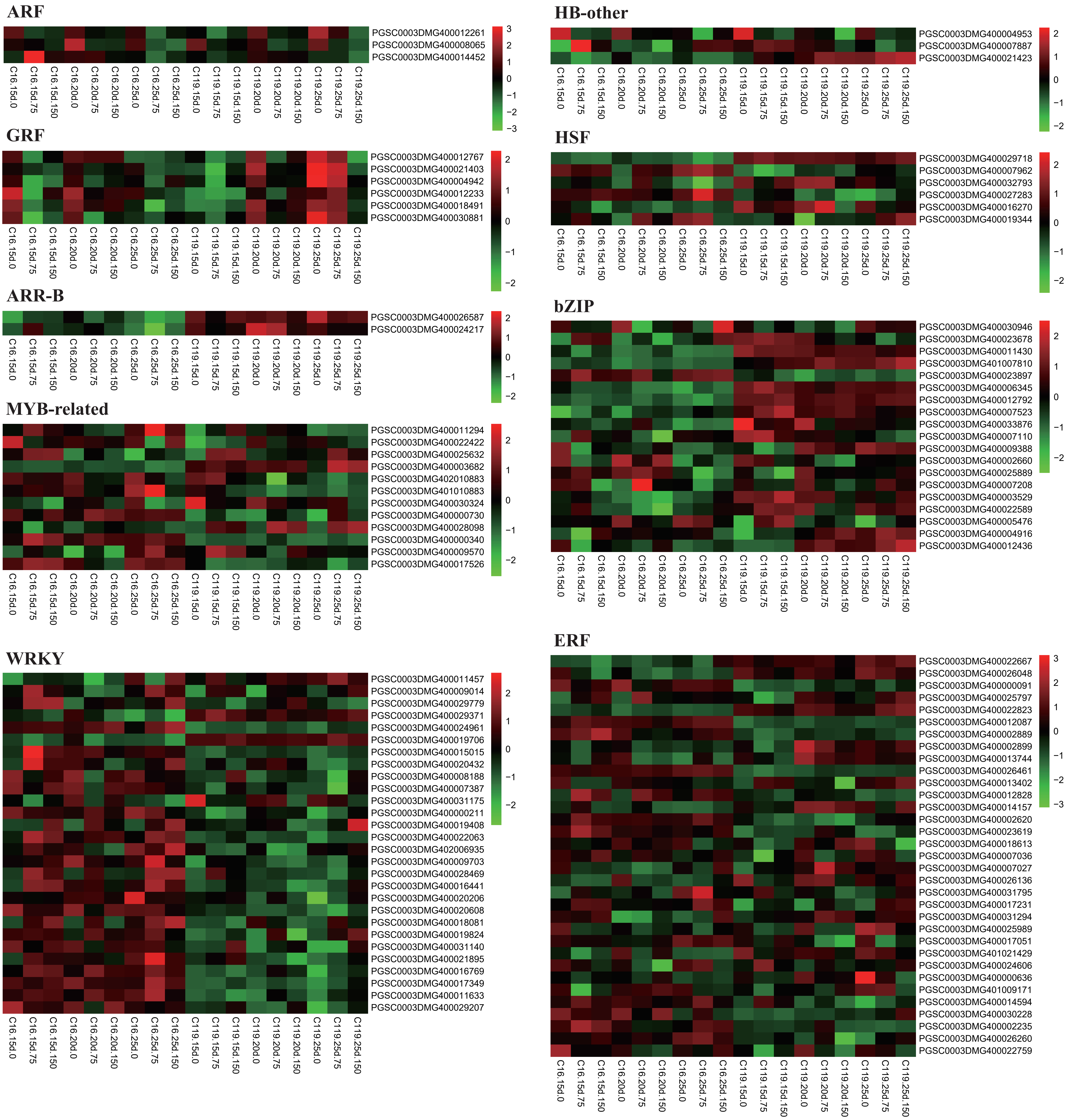

ARF
PGSC0003DMG400012261
PGSC0003DMG400008065
PGSC0003DMG400014452
3 2 1 0 −1 −2 −3
C16.15d.0 C16.15d.75 C16.15d.150 C16.20d.0 C16.20d.75 C16.20d.150 C16.25d.0 C16.25d.75 C16.25d.150 C119.15d.0 C119.15d.75 C119.15d.150 C119.20d.0 C119.20d.75 C119.20d.150 C119.25d.0 C119.25d.75 C119.25d.150
GRF
PGSC0003DMG400012767
PGSC0003DMG400021403
PGSC0003DMG400004942
PGSC0003DMG400012233
PGSC0003DMG400018491
PGSC0003DMG400030881
2 1 0 −1 −2
C16.15d.0 C16.15d.75 C16.15d.150 C16.20d.0 C16.20d.75 C16.20d.150 C16.25d.0 C16.25d.75 C16.25d.150 C119.15d.0 C119.15d.75 C119.15d.150 C119.20d.0 C119.20d.75 C119.20d.150 C119.25d.0 C119.25d.75 C119.25d.150
ARR-B
PGSC0003DMG400026587
PGSC0003DMG400024217
2 1 0 −1 −2
C16.15d.0 C16.15d.75 C16.15d.150 C16.20d.0 C16.20d.75 C16.20d.150 C16.25d.0 C16.25d.75 C16.25d.150 C119.15d.0 C119.15d.75 C119.15d.150 C119.20d.0 C119.20d.75 C119.20d.150 C119.25d.0 C119.25d.75 C119.25d.150
MYB-related
PGSC0003DMG400011294
PGSC0003DMG400022422
PGSC0003DMG400025632
PGSC0003DMG400003682
PGSC0003DMG402010883
PGSC0003DMG401010883
PGSC0003DMG400030324
PGSC0003DMG400000730
PGSC0003DMG400028098
PGSC0003DMG400000340
PGSC0003DMG400009570
PGSC0003DMG400017526
2 1 0 −1 −2
C16.15d.0 C16.15d.75 C16.15d.150 C16.20d.0 C16.20d.75 C16.20d.150 C16.25d.0 C16.25d.75 C16.25d.150 C119.15d.0 C119.15d.75 C119.15d.150 C119.20d.0 C119.20d.75 C119.20d.150 C119.25d.0 C119.25d.75 C119.25d.150
WRKY
PGSC0003DMG400011457
PGSC0003DMG400009014
PGSC0003DMG400029779
PGSC0003DMG400029371
PGSC0003DMG400024961
PGSC0003DMG400019706
PGSC0003DMG400015015
PGSC0003DMG400020432
PGSC0003DMG400008188
PGSC0003DMG400007387
PGSC0003DMG400031175
PGSC0003DMG400000211
PGSC0003DMG400019408
PGSC0003DMG400022063
PGSC0003DMG402006935
PGSC0003DMG400009703
PGSC0003DMG400028469
PGSC0003DMG400016441
PGSC0003DMG400020206
PGSC0003DMG400020608
PGSC0003DMG400018081
PGSC0003DMG400019824
PGSC0003DMG400031140
PGSC0003DMG400021895
PGSC0003DMG400016769
PGSC0003DMG400017349
PGSC0003DMG400011633
PGSC0003DMG400029207
2 1 0 −1 −2
C16.15d.0 C16.15d.75 C16.15d.150 C16.20d.0 C16.20d.75 C16.20d.150 C16.25d.0 C16.25d.75 C16.25d.150 C119.15d.0 C119.15d.75 C119.15d.150 C119.20d.0 C119.20d.75 C119.20d.150 C119.25d.0 C119.25d.75 C119.25d.150
HB-other
PGSC0003DMG400004953
PGSC0003DMG400007887
PGSC0003DMG400021423
2 1 0 −1 −2
C16.15d.0 C16.15d.75 C16.15d.150 C16.20d.0 C16.20d.75 C16.20d.150 C16.25d.0 C16.25d.75 C16.25d.150 C119.15d.0 C119.15d.75 C119.15d.150 C119.20d.0 C119.20d.75 C119.20d.150 C119.25d.0 C119.25d.75 C119.25d.150
HSF
PGSC0003DMG400029718
PGSC0003DMG400007962
PGSC0003DMG400032793
PGSC0003DMG400027283
PGSC0003DMG400016270
PGSC0003DMG400019344
2 1 0 −1 −2
C16.15d.0 C16.15d.75 C16.15d.150 C16.20d.0 C16.20d.75 C16.20d.150 C16.25d.0 C16.25d.75 C16.25d.150 C119.15d.0 C119.15d.75 C119.15d.150 C119.20d.0 C119.20d.75 C119.20d.150 C119.25d.0 C119.25d.75 C119.25d.150
bZIP
PGSC0003DMG400030946
PGSC0003DMG400023678
PGSC0003DMG400011430
PGSC0003DMG401007810
PGSC0003DMG400023897
PGSC0003DMG400006345
PGSC0003DMG400012792
PGSC0003DMG400007523
PGSC0003DMG400033876
PGSC0003DMG400007110
PGSC0003DMG400009388
PGSC0003DMG400002660
PGSC0003DMG400025889
PGSC0003DMG400007208
PGSC0003DMG400003529
PGSC0003DMG400022589
PGSC0003DMG400005476
PGSC0003DMG400004916
PGSC0003DMG400012436
2 1 0 −1 −2
C16.15d.0 C16.15d.75 C16.15d.150 C16.20d.0 C16.20d.75 C16.20d.150 C16.25d.0 C16.25d.75 C16.25d.150 C119.15d.0 C119.15d.75 C119.15d.150 C119.20d.0 C119.20d.75 C119.20d.150 C119.25d.0 C119.25d.75 C119.25d.150
ERF
PGSC0003DMG400022667
PGSC0003DMG400026048
PGSC0003DMG400000091
PGSC0003DMG400025797
PGSC0003DMG400022823
PGSC0003DMG400012087
PGSC0003DMG400002889
PGSC0003DMG400002899
PGSC0003DMG400013744
PGSC0003DMG400026461
PGSC0003DMG400013402
PGSC0003DMG400012828
PGSC0003DMG400014157
PGSC0003DMG400002620
PGSC0003DMG400023619
PGSC0003DMG400018613
PGSC0003DMG400007036
PGSC0003DMG400007027
PGSC0003DMG400026136
PGSC0003DMG400031795
PGSC0003DMG400017231
PGSC0003DMG400031294
PGSC0003DMG400025989
PGSC0003DMG400017051
PGSC0003DMG401021429
PGSC0003DMG400024606
PGSC0003DMG400000636
PGSC0003DMG401009171
PGSC0003DMG400014594
PGSC0003DMG400030228
PGSC0003DMG400002235
PGSC0003DMG400026260
PGSC0003DMG400022759
3 2 1 0 −1 −2 −3
C16.15d.0 C16.15d.75 C16.15d.150 C16.20d.0 C16.20d.75 C16.20d.150 C16.25d.0 C16.25d.75 C16.25d.150 C119.15d.0 C119.15d.75 C119.15d.150 C119.20d.0 C119.20d.75 C119.20d.150 C119.25d.0 C119.25d.75 C119.25d.150
